# Supplementary material for: Novel neuromuscular controllers with simplified muscle model and enhanced reflex modulation: A comparative study in hip exoskeletons
Source: Wearable Technol. 2024 Dec 10;5:e21. doi: 10.1017/wtc.2024.11 (PMC11729522; doi:10.1017/wtc.2024.11)
Supplement: Manzoori et al. supplementary material [file S2631717624000112sup001.pdf]

Wearable Technologies (2024), **xx**:xx 1–15
doi:10.1017/xxxx

SUPPLEMENTARY MATERIAL

Supplementary Material for: Novel Neuromuscular Controllers with Simplified Muscle Model and Enhanced Reflex Modulation: A Comparative Study in Hip Exoskeletons

Ali Reza Manzoori[*1], Sara Messara[*1], Andrea Di Russo[1], Auke Ijspeert[1] and Mohamed Bouri[1,2]

[*]These two authors contributed equally to this work and are designated as co-first authors. [1]Biorobotics Laboratory, EPFL, Lausanne, 1015, Vaud, Switzerland. [2]Translational Neural Engineering Laboratory, EPFL, Lausanne, 1015, Vaud, Switzerland.

(Received xx xxx xxxx)

Supplementary Table 1. List of abbreviations and acronyms used in the article.

Abbreviation/Acronym	Description	Abbreviation/Acronym	Description
BE	Buffer element	MS	Mid-stance
CE	Contractile element	MTU	Muscle-tendon unit
ES	Early stance	NLP	Nonlinear passive element
FSM	Finite-state machine	NMC	Neuromuscular controller
GLU	Gluteus maximus	PE	Parallel element
HIL	human in the loop	PS	Pre-swing
ILPS	Illiopsoas	S	Swing
LP	Landing preparation	SE	Serial element

Supplementary Table 2. List of the symbols used to denote the parameters and variables in the NMC equations.

Symbol	Description	Symbol	Description
Act	Muscle activation signal	ℓ_{PE}	PE length
C	Constant inhibition term for muscle stimulation	$\ell_{\mathrm{SE,slack}}$	SE slack length
δt	Neural signal propagation time constant	ℓ_{SE}	SE length
ϵ_{BE}	BE length	ℓ_{slack}	SE (tendon) slack length
ϵ_{PE}	PE length	N	Normalized MTU force, defined as $F_{\mathrm{CE}}/F_{\mathrm{max}}$
ϵ_{SE}	SE length	ω	Constant determining the Gaussian width in the force-length relationship
F_{BE}	BE force	P_i	Generic notation for the reflex sensory inputs
F_{max}	Maximum MTU isometric force	r	MTU moment arm around the joint
F_{MTU}	MTU force	r_0	Moment arm constant
F_{PE}	PE force	ρ	Muscle pennation angle constant

Continued on the next page...

© Cambridge University Press 2019. This is an Open Access article, distributed under the terms of the Creative Commons Attribution licence (http://creativecommons.org/licenses/by/4.0/), which permits unrestricted re-use, distribution, and reproduction in any medium, provided the original work is properly cited.

***Supplementary Table 2** – continued from previous page.*

Symbol	Description	Symbol	Description
F_{SE}	SE force	$Stim$	Muscle stimulation signal
f_ℓ	Force-length relationship	$Stim_0$	Basal stimulation level
f_v	Force-velocity relationship	t	Time
$G_{s,v}$	Manually adjusted assistive torque scaling gain	T_{assist}	Total assistive torque generated by the NMC
G_i	Generic notation for the reflex gains	T_{GLU}	Torque generated by the (virtual) gluteus maximus around the hip joint
G_ℓ	Length reflex gain	T_{ILPS}	Torque generated by the (virtual) illiopsoas around the hip joint
K	Curvature constant for the force-velocity relationship	T_{MTU}	Torque generated by a certain MTU around the corresponding joint
$\ell_{BE,slack}$	BE slack length	τ	Muscle stimulation-to-activation time constant
ℓ_{BE}	BE length	θ	Joint angle
ℓ_{CE}	CE length	θ_{ref}	Reference joint angle at which $\ell_{\mathrm{MTU}} = \ell_{\mathrm{opt}} + \ell_{\mathrm{slack}}$
ℓ_{MTU}	MTU length	v_{CE}	CE force
ℓ_{opt}	Optimum CE length	v_{max}	Maximum CE contraction velocity in the force-velocity relationship
$\ell_{\mathrm{PE,slack}}$	PE slack length		

Supplementary Table 3. Neuromuscular model hip joint parameter values

Parameter [Unit]	GLU value	ILPS value
$\ell_{\mathrm{slack}}\,[m]$	0.1	0.1
$\ell_{\mathrm{opt}}\,[m]$	0.157	0.13
$\theta_{\mathrm{ref}}\,[deg]$	170.0	180.0
$F_{\mathrm{max}}\,[N]$	1500	1500
$v_{\mathrm{max}}\,[N]$	1.32	1.32
$\tau\,[s]$	0.1	0.01
$\omega\,[]$	0.56	0.56
$\theta_{\mathrm{max}}\,[deg]$	0	0
$\theta_{\mathrm{off}}\,[deg]$	0	0
$r_0\,[m]$	0.1	0.1
$\rho\,[]$	0.5	0.5
$\ell_{\mathrm{off}}\,[m]$	0.157	0.157
$K\,[]$	0.005	0.005
$N\,[]$	1.5	1.5
$C\,[]$	0.05	0.05
$Stim_0\,[]$	0.01	0.01
$\delta t\,[s]$	0.25	0.25

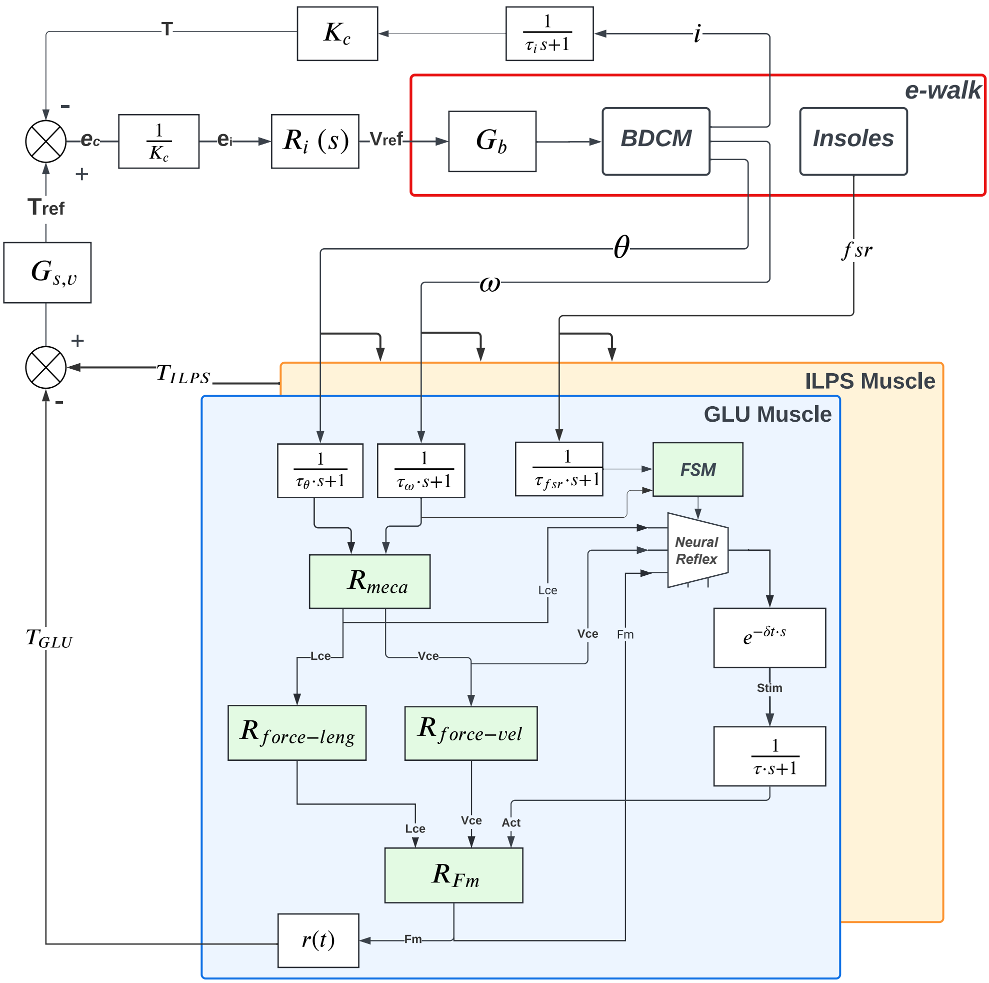

Supplementary Figure 1. Detailed block diagram of the novel NMC architecture, as implemented on the e-Walk V1 hip exoskeleton. R_{meca} is the set of the mechanical equations mapping the hip angle to the CE length (as in 14) and the hip angular velocity to the CE velocity (as in 15); $R_{force-leng}$ is the force-length relationship given in equation 6; $R_{force-vel}$ is the force-velocity relationship as in 7; the low-pass filters with time constants τ_θ, τ_ω and τ_{fsr} were added to reduce sensor noise from the angle, angular velocity and the insole pressure signals, respectively; R_{Fm} gives the MTU force as in equation 18; the *FSM* block updates the gait phase according to the inputs; the $Reflex$ multiplexer represents the selection of the proper neural reflex; the exponential term represents the stimulation's phase delay of time δt; the low-pass filter with constant time τ represents the activation-stimulation relationship as in 5.

Supplementary Table 4. Mean positive (W+) and negative (W-) mechanical works per stride delivered by the exoskeleton (left side only), averaged over all participants and strides under each condition.

Condition	NMC-4		NMC-5	
	W+ [J/kg]	W- [J/kg]	W+ [J/kg]	W- [J/kg]
**C1 (0.8 m/s)**	0.122	-0.010	0.074	-0.003
**C2 (1.25 m/s)**	0.162	-0.013	0.103	-0.003
**C3 (1.8 m/s)**	0.195	-0.015	0.142	-0.004
**C4 (1.25 m/s, 10%)**	0.154	-0.007	0.137	0.000

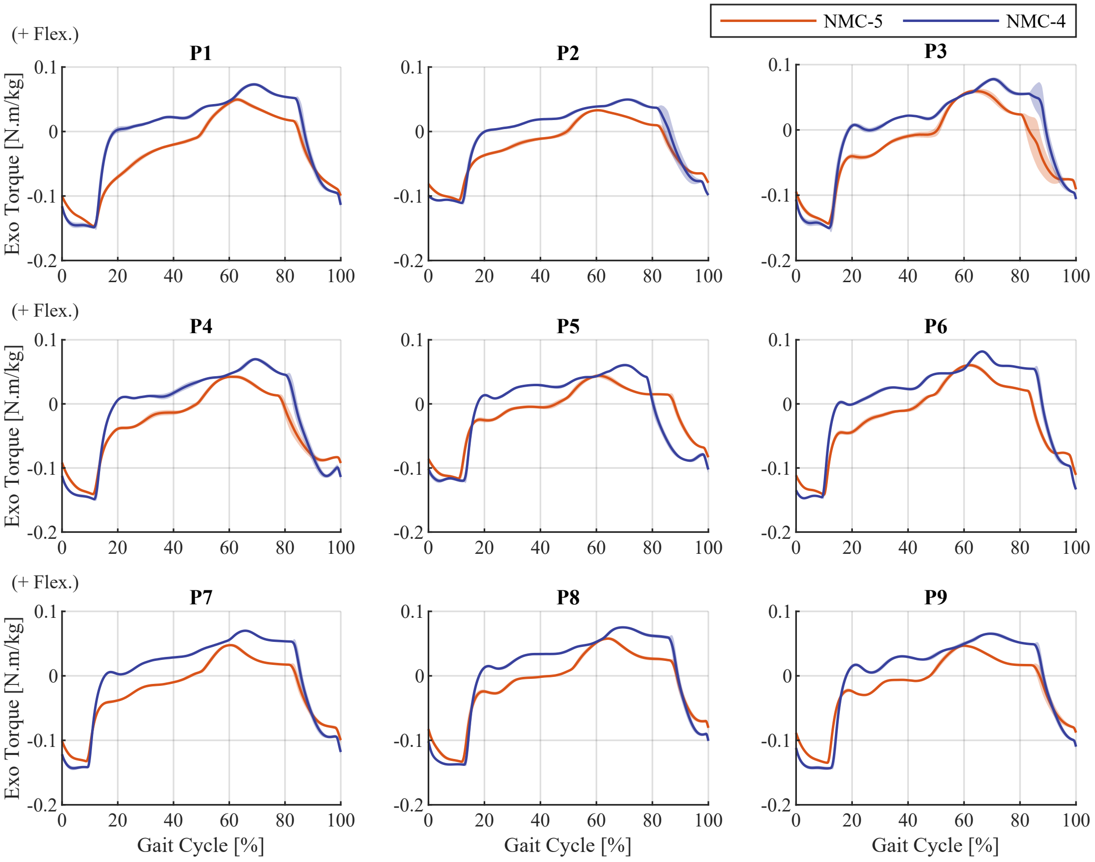

Supplementary Figure 2. Torque profiles generated by the four-state (NMC-4) and five-state (NMC-5) variants under condition C1 for all participants.

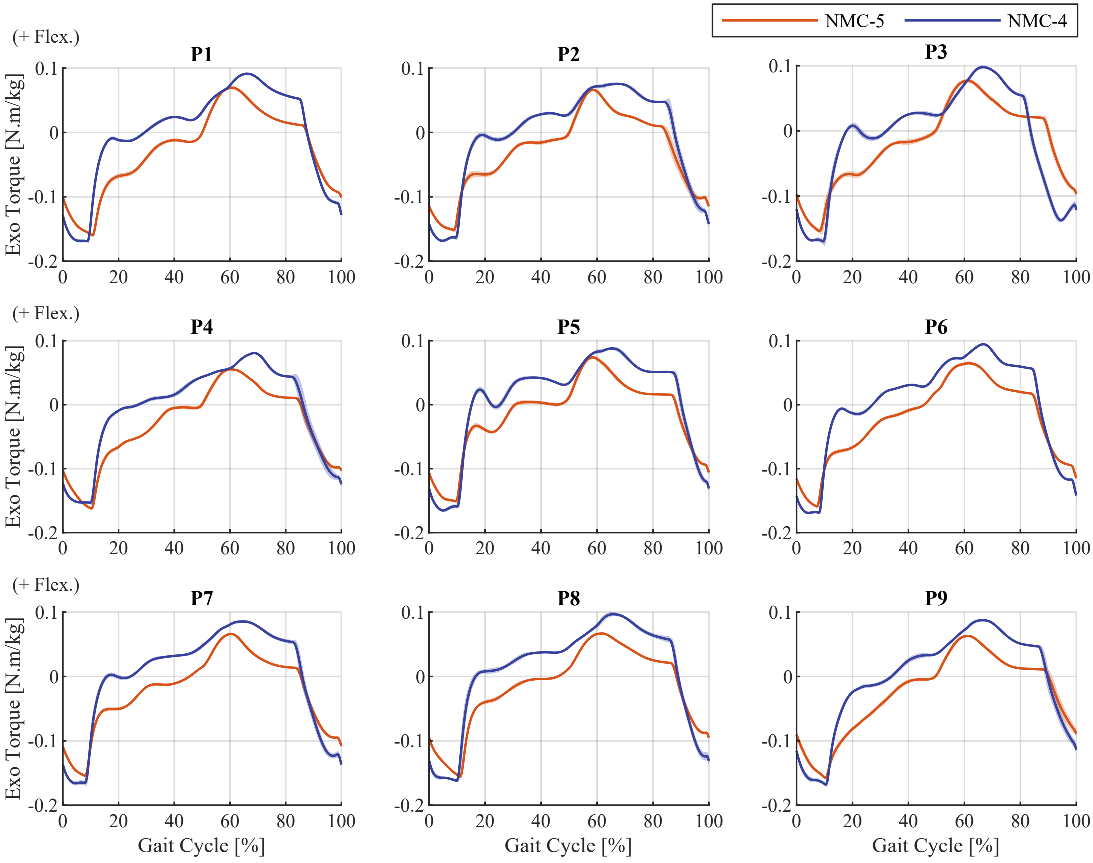

Supplementary Figure 3. Torque profiles generated by the four-state (NMC-4) and five-state (NMC-5) variants under condition C2 for all participants.

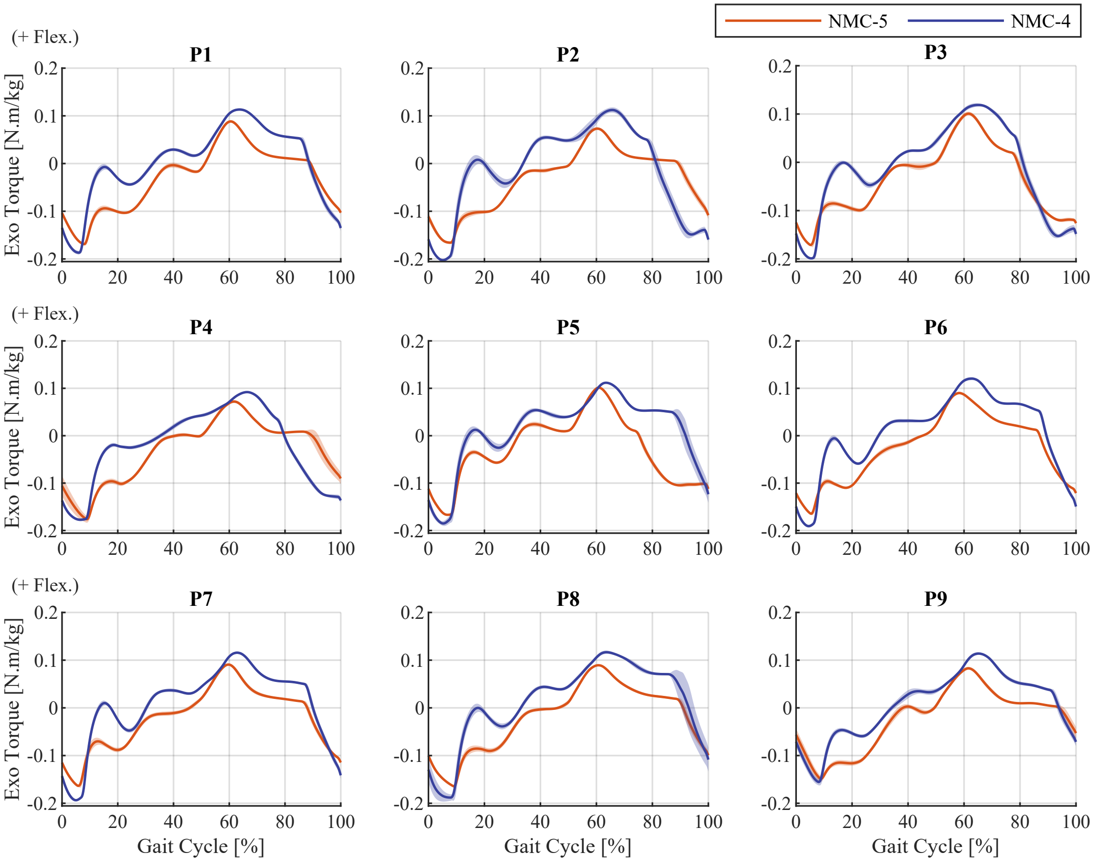

Supplementary Figure 4. Torque profiles generated by the four-state (NMC-4) and five-state (NMC-5) variants under condition C3 for all participants.

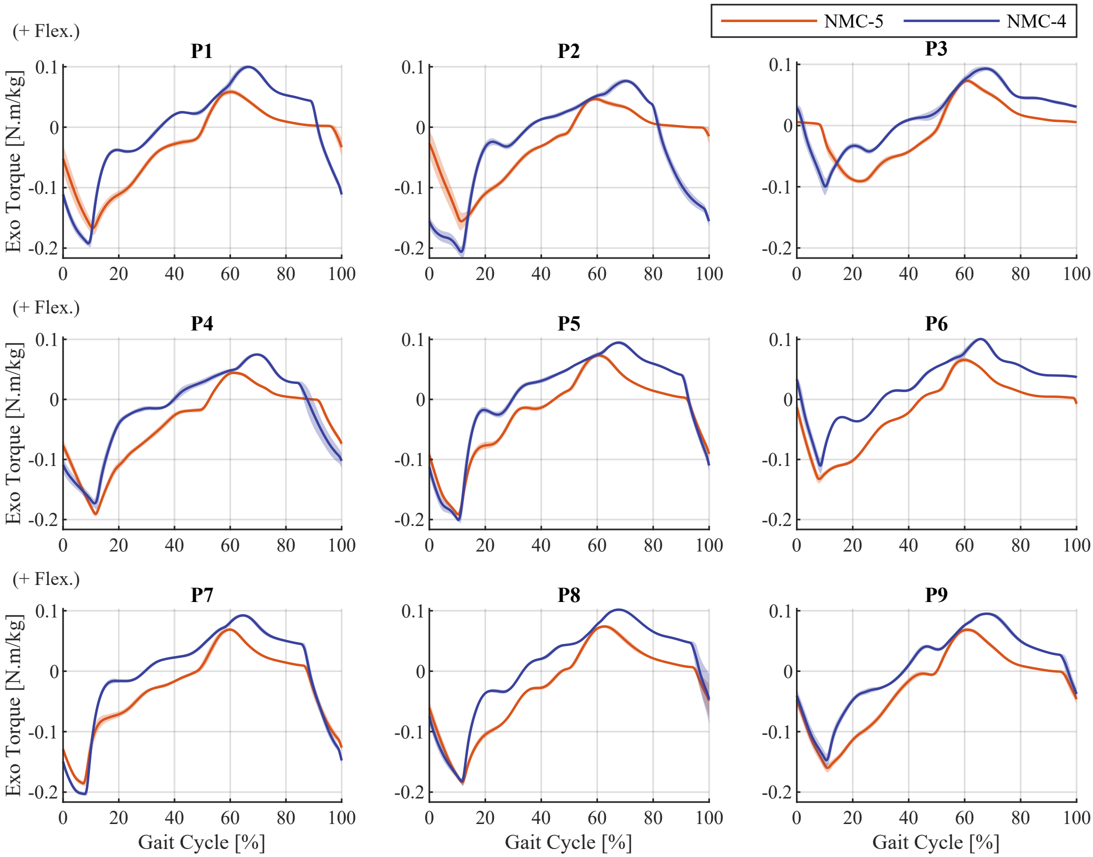

Supplementary Figure 5. Torque profiles generated by the four-state (NMC-4) and five-state (NMC-5) variants under condition C4 for all participants.

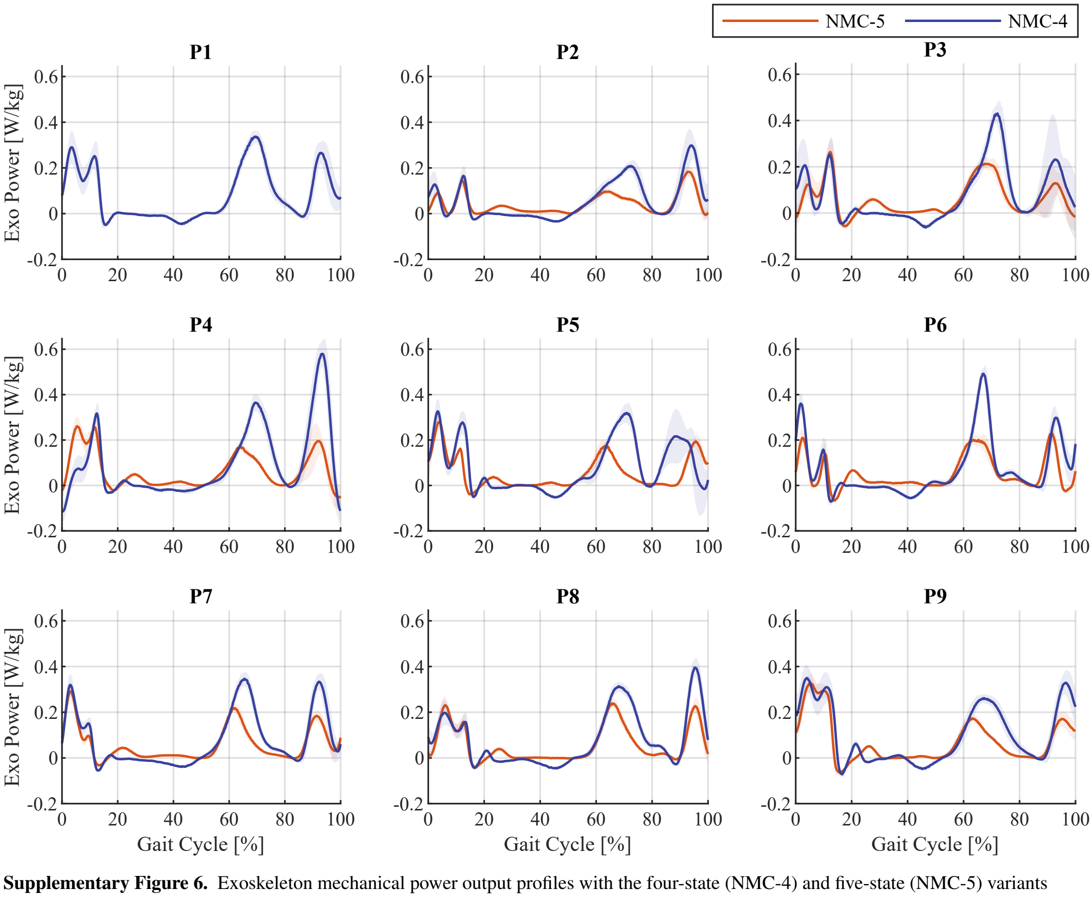

Supplementary Figure 6. Exoskeleton mechanical power output profiles with the four-state (NMC-4) and five-state (NMC-5) variants under condition C1 for all participants. Note that for participant P1, due to an issue in the logging system, the power profile for NMC-5 was not available.

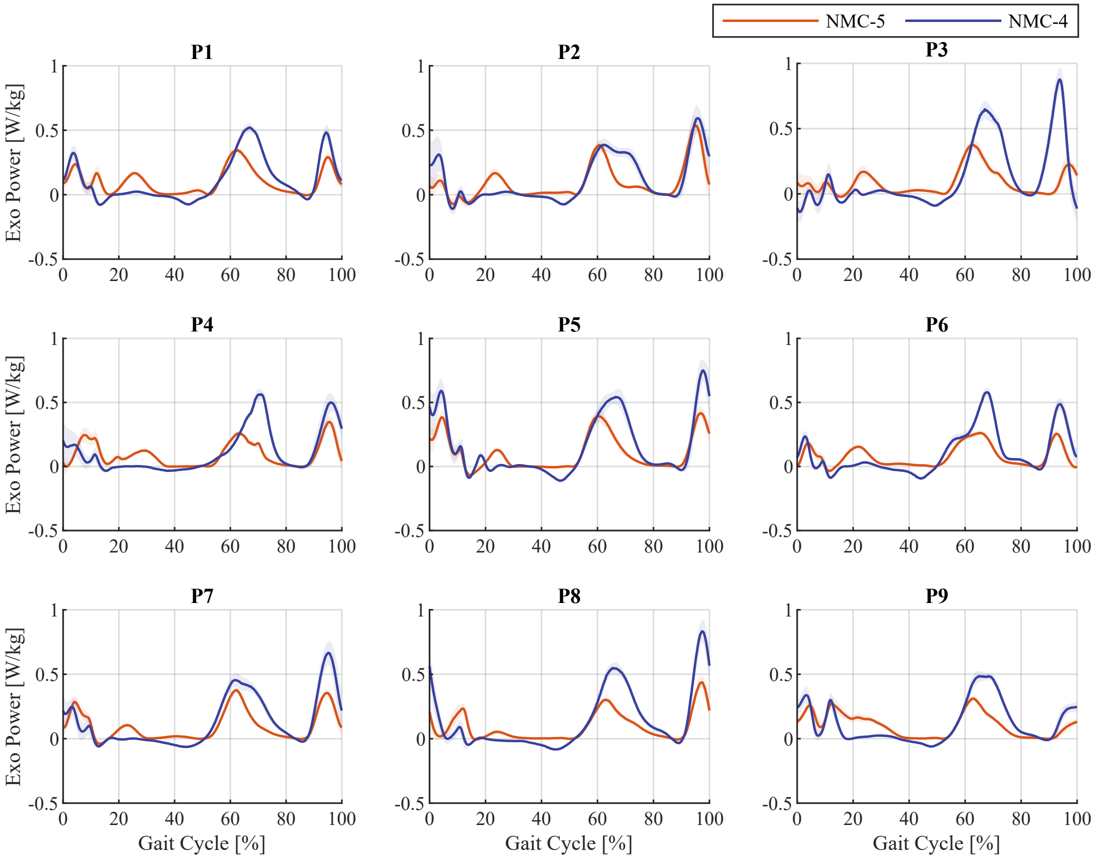

Supplementary Figure 7. Exoskeleton mechanical power output profiles with the four-state (NMC-4) and five-state (NMC-5) variants under condition C2 for all participants.

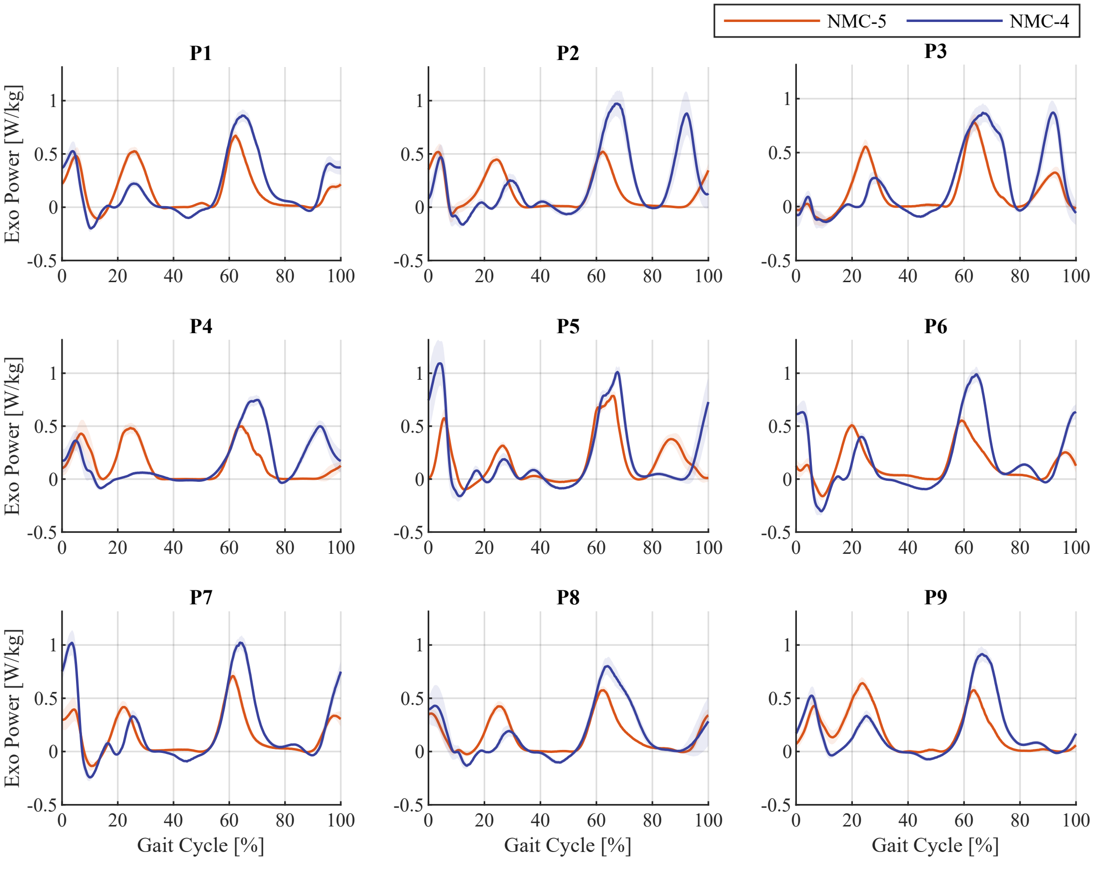

Supplementary Figure 8. Exoskeleton mechanical power output profiles with the four-state (NMC-4) and five-state (NMC-5) variants under condition C3 for all participants.

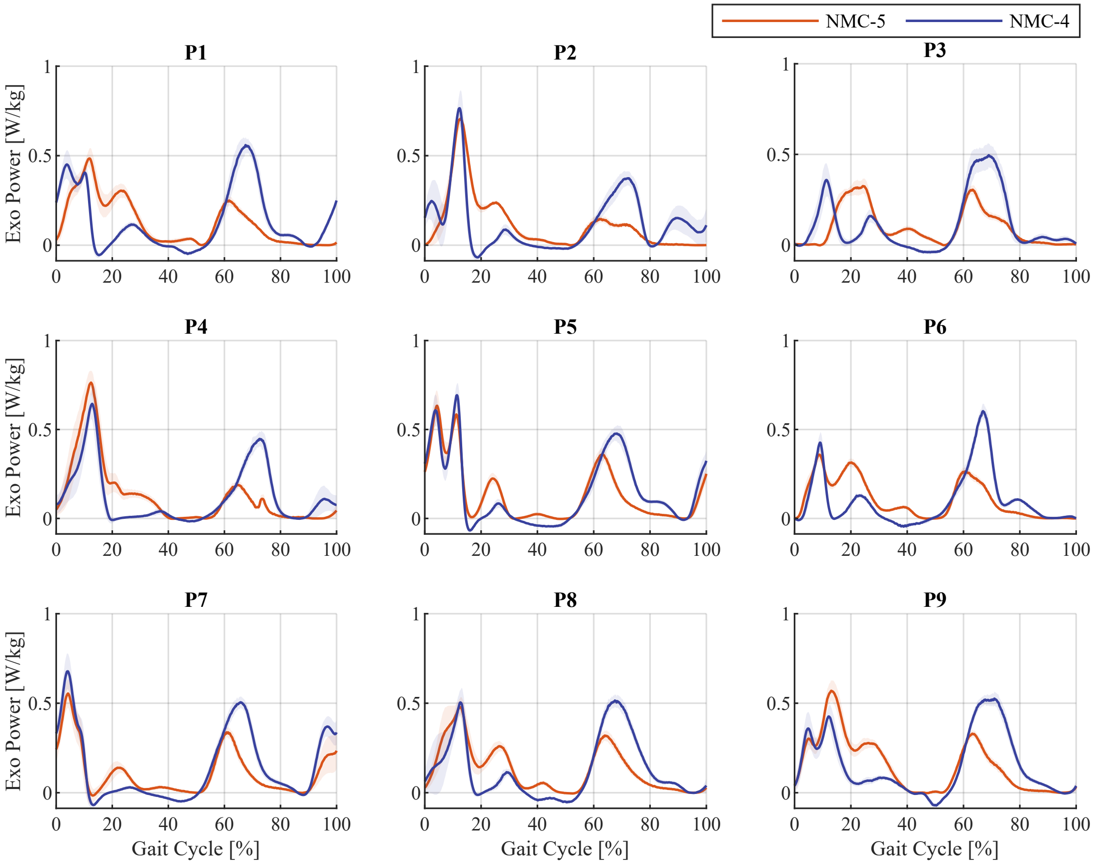

Supplementary Figure 9. Average exoskeleton mechanical power output profiles with the four-state (NMC-4) and five-state (NMC-5) variants under condition C4 for all participants.

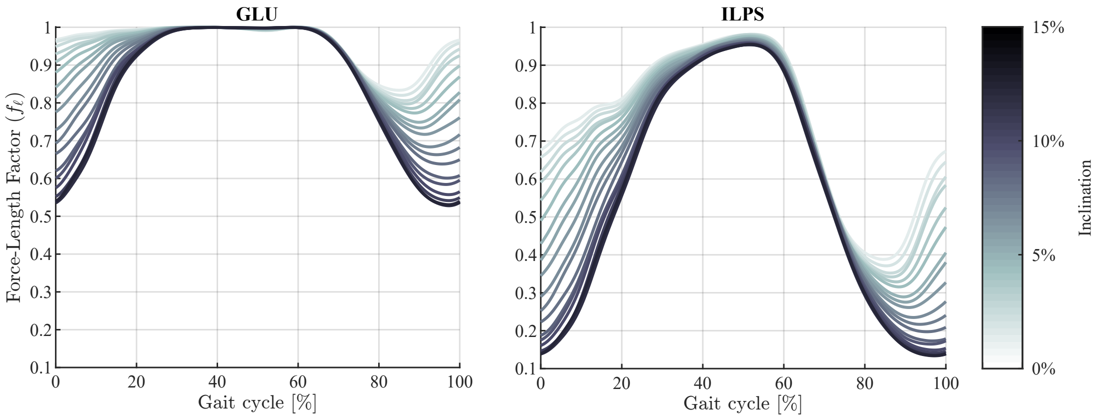

Supplementary Figure 10. Evolution of the force-length factor (f_ℓ) for the extensor (gluteus maximus, Glu) and flexor (illiopsoas, Ilps) virtual muscles in NMC-5 during variable-inclination walking (C5), averaged over the six valid participants.

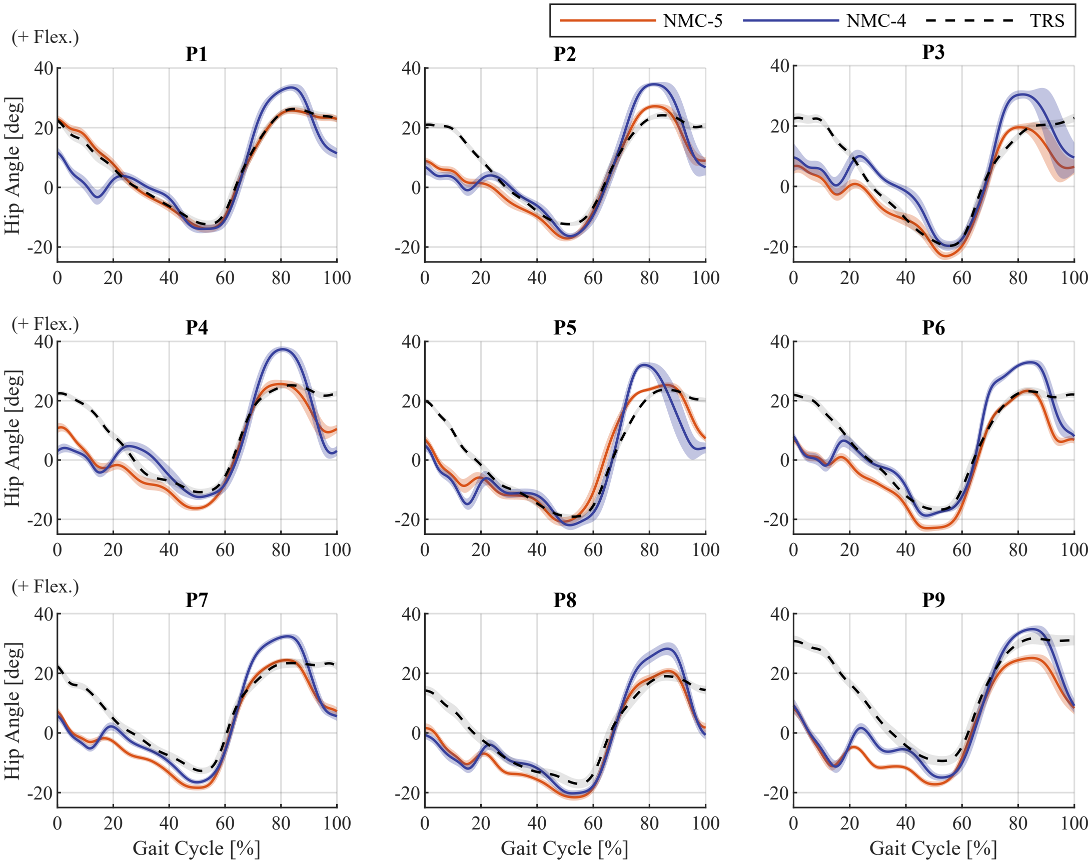

Supplementary Figure 11. Average hip angle profiles in assisted walking (NMC-4 and NMC-5) and unassisted mode (TRS) for all participants under condition C1.

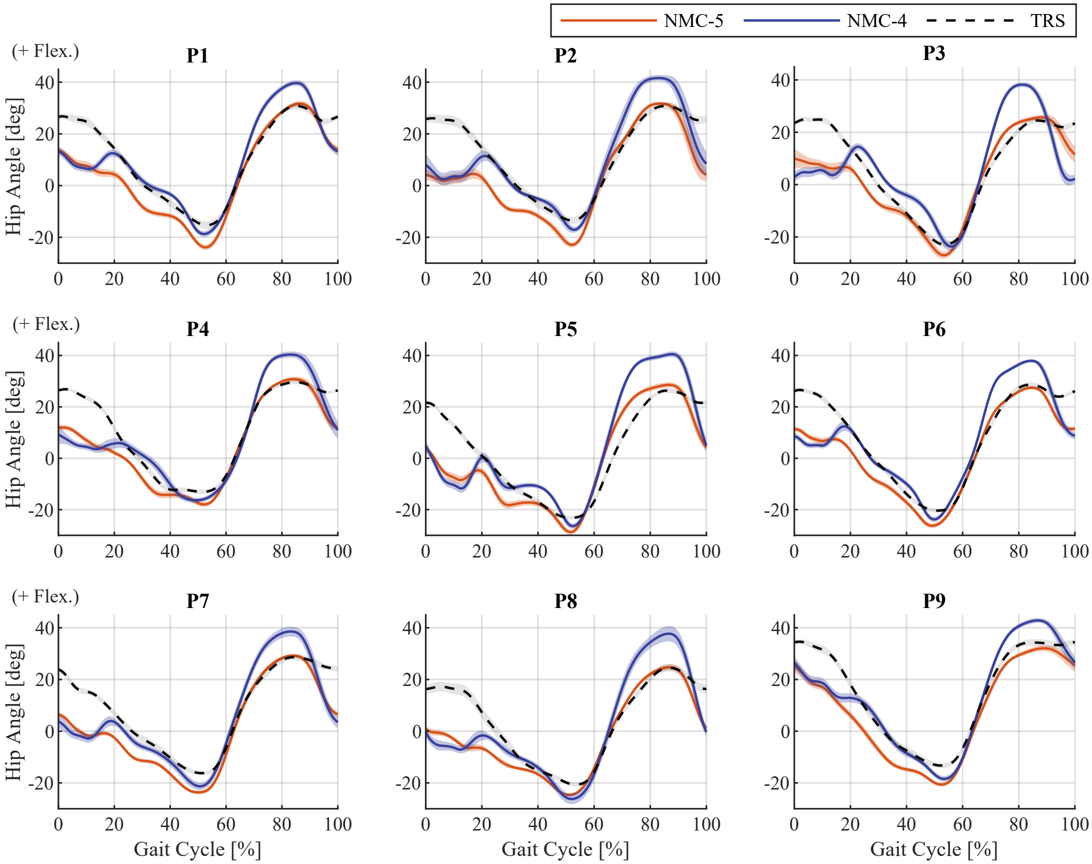

Supplementary Figure 12. Average hip angle profiles in assisted walking (NMC-4 and NMC-5) and unassisted mode (TRS) for all participants under condition C2.

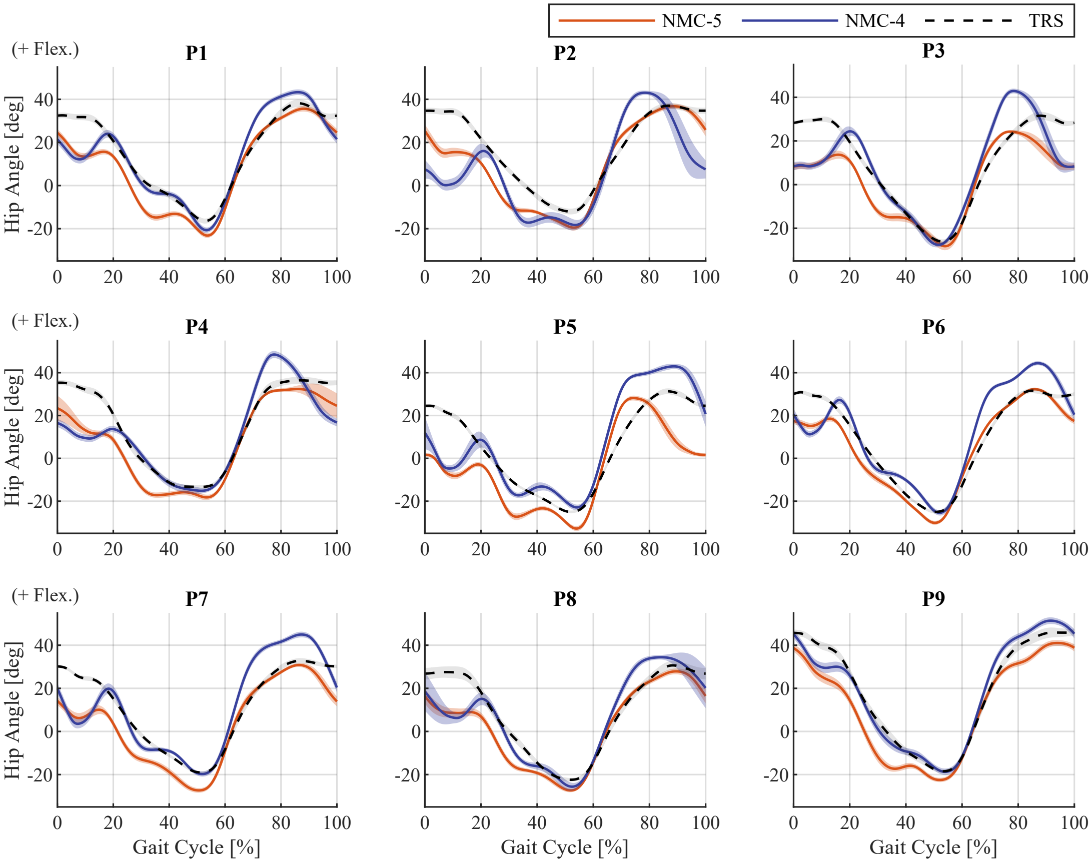

Supplementary Figure 13. Average hip angle profiles in assisted walking (NMC-4 and NMC-5) and unassisted mode (TRS) for all participants under condition C3.

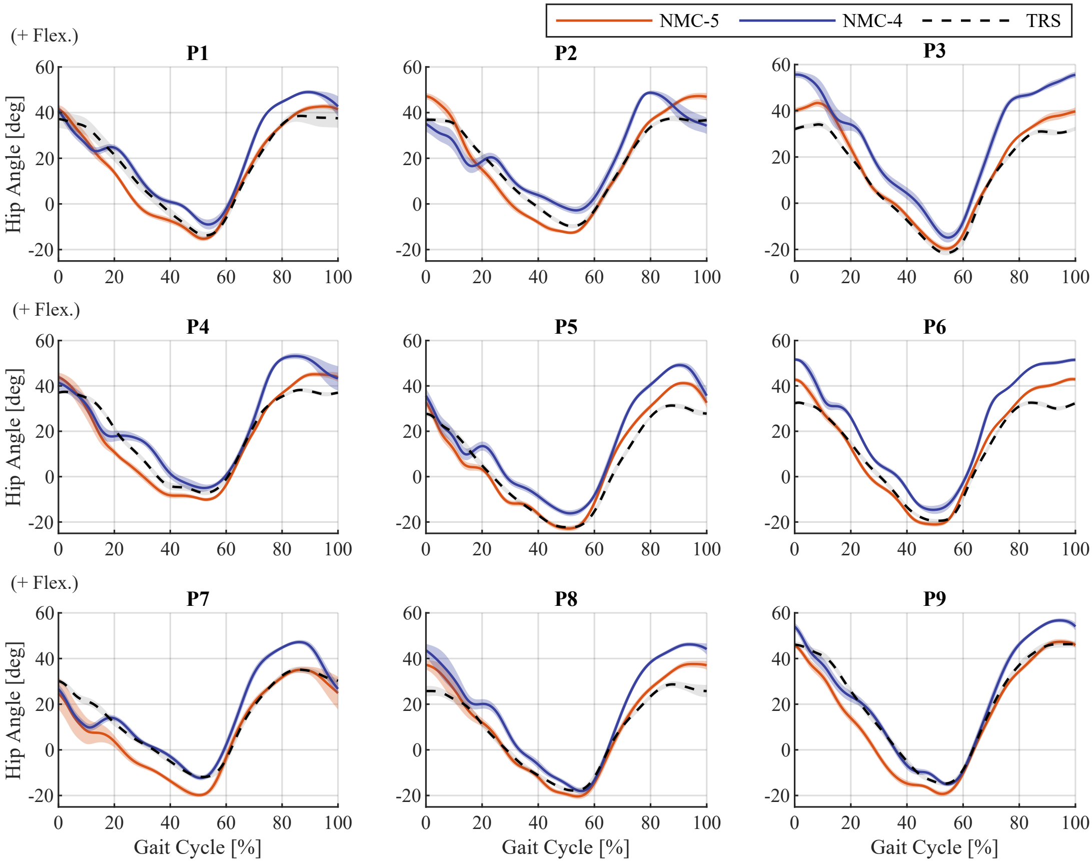

Supplementary Figure 14. Average hip angle profiles in assisted walking (NMC-4 and NMC-5) and unassisted mode (TRS) for all participants under condition C4.
